# Detection of Talking in Respiratory Signals: A Feasibility Study Using Machine Learning and Wearable Textile-Based Sensors

**DOI:** 10.3390/s18082474

**Published:** 2018-07-31

**Authors:** Andreas Ejupi, Carlo Menon

**Affiliations:** Menrva Research Group, Schools of Mechatronic Systems & Engineering Science at Simon Fraser University (SFU), Burnaby, BC V5A 1S6, Canada; andreas@ejupi.at

**Keywords:** wearable sensors, machine learning, smart textiles, healthcare, talking detection

## Abstract

Social isolation and loneliness are major health concerns in young and older people. Traditional approaches to monitor the level of social interaction rely on self-reports. The goal of this study was to investigate if wearable textile-based sensors can be used to accurately detect if the user is talking as a future indicator of social interaction. In a laboratory study, fifteen healthy young participants were asked to talk while performing daily activities such as sitting, standing and walking. It is known that the breathing pattern differs significantly between normal and speech breathing (i.e., talking). We integrated resistive stretch sensors into wearable elastic bands, with a future integration into clothing in mind, to record the expansion and contraction of the chest and abdomen while breathing. We developed an algorithm incorporating machine learning and evaluated its performance in distinguishing between periods of talking and non-talking. In an intra-subject analysis, our algorithm detected talking with an average accuracy of 85%. The highest accuracy of 88% was achieved during sitting and the lowest accuracy of 80.6% during walking. Complete segments of talking were correctly identified with 96% accuracy. From the evaluated machine learning algorithms, the random forest classifier performed best on our dataset. We demonstrate that wearable textile-based sensors in combination with machine learning can be used to detect when the user is talking. In the future, this approach may be used as an indicator of social interaction to prevent social isolation and loneliness.

## 1. Introduction

Social isolation and loneliness are important health risk factors and known to negatively influence wellbeing. It has been reported that up to 50% of older people suffer from a low level of social interaction [1]. The causes can be diverse including general health issues, disabilities and certain life events such as the loss of a spouse or a change in residence [2,3]. On a positive note, research has shown that social isolation and loneliness can be prevented. Intervention programs such as in-person support activities or phone-mediated groups have shown promising results [3]. However, due to the limited health care resources, it would be warranted to accurately identify people who are in need of targeted interventions. Traditional approaches rely on the self-reports using questionnaires to assess the daily level of social interaction. Self-reports are often described as subjective and influenced by a recall bias [4].

One alternative approach could be to automatically identify people with a low level of social interaction by using technology. Previous work in this area has mainly focused on audio-based systems using a microphone to capture talking throughout the day [5,6]. Previous work has also investigated the use of video-based systems to monitor mouth movements as an indicator of social interaction [7,8]. Both methods look promising in terms of accuracy. However, user acceptance and portability might be a challenge [9].

There is a need for more unobtrusive and portable solutions. We propose to detect if someone is talking by using wearable textile-based sensors, which can be directly integrated into everyday clothing. Our approach does not rely on audio or video recordings; instead, it aims to detect talking by monitoring changes in the respiratory (i.e., breathing) patterns.

### 1.1. Detection of Talking (Speech Breathing)

Generally, breathing results in an expansion and a contraction of the chest and abdominal region. It has been found that the breathing pattern differs significantly between normal and speech breathing (i.e., talking), with the respiration more rhythmic during normal breathing [10,11]. It has been also reported that the inhalation duration and the ratio between the inhalation and exhalation time are good discriminatory indicators [12,13].

To date, only a few studies have investigated the use of wearable sensors to detect if someone is talking based on respiratory markers [10,12,14]. These studies used inductive plethysmography sensors, which consist of electrical wires embedded in elastic bands usually attached to the chest and abdominal region. By generating a magnetic field and passing it through a sinusoidal arrangement of electrical wires, the self-conductance of the coils, which is proportional to the cross-sectional area surrounded by the band, can be measured [12]. However, these sensors are primarily designed for clinical settings and mainly used for short duration recordings.

### 1.2. Textile-Based Sensors

In this paper, we investigate the feasibility of wearable textile-based sensors. In particular, we focus on resistive stretch sensors, which are made by a mixture of polymer (e.g., silicone, rubber) and a conductive material (e.g., carbon black). These resistive sensors act like a resistor, which means that any elongation results in a measurable change in electrical resistance. Related work in this field has investigated the use of textile-based stretch sensors in several human applications. For example, Tognetti et al. [15] investigated a textile-based sensor for posture monitoring. Similarly, Mattman et al. [16] integrated sensors into tight-fitting clothing to classify between various body postures. Papi et al. [17] explored the feasibility to discriminate between daily activities (i.e., walking, running, stair climbing) by using a stretch sensor attached to the knee. These studies suggest the preliminary feasibility of textile-based stretch sensors to monitor human motions. To the best of our knowledge, our study is the first to use this type of sensor to detect talking in respiratory signals.

The main aims of this study were to (1) investigate the feasibility of textile-based stretch sensors to monitor breathing patterns, (2) develop an algorithm using machine learning to accurately detect talking and (3) evaluate its performance in a study with 15 participants.

## 2. Materials and Methods

### 2.1. Stretch Sensor

**Figure 1:**
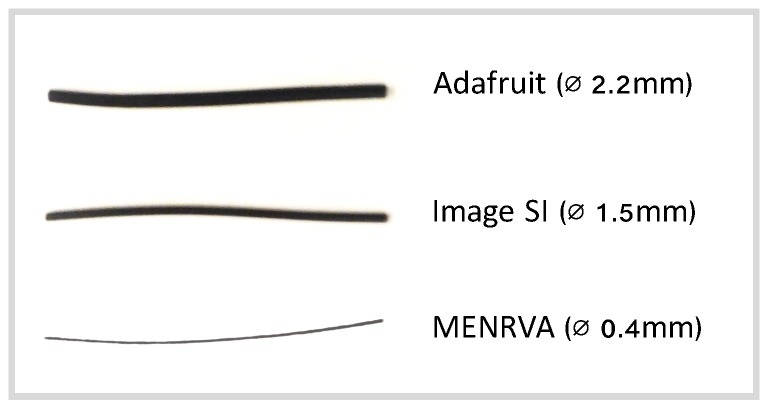
Comparison between the Adafruit, Image SI and Menrva sensors.

In this paper, we investigated the feasibility of a wearable textile-based stretch sensor to detect if someone is talking. The stretch sensor has been fabricated in our research lab (Menrva) at Simon Fraser University, Canada [18], using a mixture of polymer and conductive carbon black. The sensor shows similar properties as the commercially available sensors from Adafruit (New York, NY, USA) [19] and Image SI (Staten Island, NY, USA) [20], but only has a diameter of 0.4 mm, which makes it suitable to integrate into garments (Figure 1). Previous work has shown good results in using machine learning to obtain accurate measurements from these textile-based stretch sensors [21,22] and using them for the monitoring of human movements [15,16,23].

### 2.2. Chest and Abdominal Bands

The approach was to detect talking based on changes in the breathing pattern. As is known from the literature, we can differentiate between chest and abdominal breathing [24,25]. Chest breathing can be described as the drawing of air into the chest area by using the intercostal muscles. This type of breathing is more common during states of exertion. In contrast, abdominal breathing is the expansion of the belly by contracting the diaphragm. This type of breathing is common during phases of relaxation [26].

**Figure 2:**
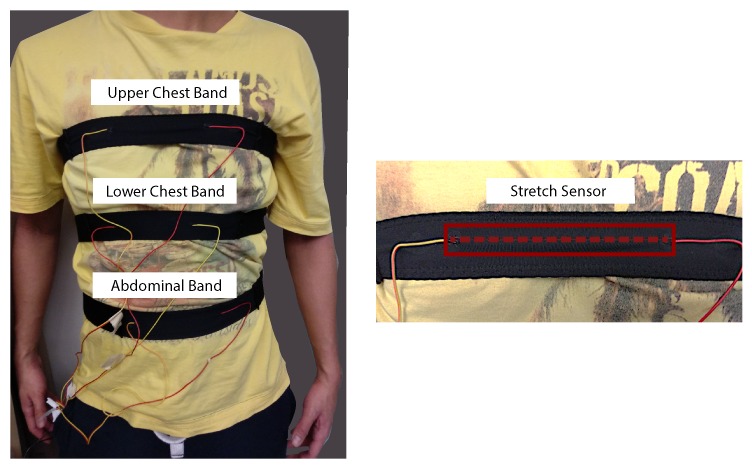
Three custom-made sensor bands to monitor the expansion and contraction of the torso while breathing (and talking). The red dashed line shows the positioning of the sensor.

However, breathing can be quite diverse between people. Some people are more heavily chest breathers, whereas others are more so abdominal breathers [25]. To capture the expansion and contraction of the full torso, we designed three elastic bands with the stretch sensor integrated and positioned them at the abdominal, lower and upper chest region for our study (Figure 2). In the future, the sensor might be directly integrated into the clothing.

The bands were made out of two materials. The back and side part were made out of a synthetic knit with medium elasticity. The front piece and attachment of the sensor were made of a fleece material with high elasticity. The intention was to concentrate the stretch during breathing (and talking) primarily on the sensor. Three pieces of the Menrva stretch sensor with a length of 10 cm each were integrated into the front piece of the bands (Figure 2). Sensors were laid out straight and secured with an elastic stitch on top. The wires were connected on both sides with a mixture of rubber glue and conductive ink.

### 2.3. Data Acquisition Hardware

The three bands were connected to a data acquisition system (Model NI-USB-6009, National Instruments, Austin, TX, USA) using a voltage divider circuit to measure their electrical response by connecting a 5 V DC voltage source and a resistor in series to the sensors. The resistor value was selected to match the base resistance of the stretch sensor (20 kΩ). All data were captured with a sampling rate of 100 Hz.

### 2.4. Study Protocol

The study protocol included three main parts with a total duration of 1.5 h per participant including the setup time. Participants were asked to wear the three custom-made sensors to monitor the expansion and contraction of the torso while talking. Sensor bands were tightly fitted, but still comfortable, for each participant. The tightness was adjusted based on the user’s feedback by explaining that the bands should be similarly tight and comfortable as, for example, a tight-fitting t-shirt, usually used for exercising. Participants were asked to talk while sitting, standing and walking. We selected these activities because they are the most common activities in which people talk in daily life. Each activity lasted for 20 min and included 5 trials with 2 min of non-talking and 2 min of talking. The order of the activities was randomized. To capture sufficient data of talking during each period and activity, we asked the participants to read out the text of a news article. The article included general information about the city of Vancouver, Canada. For the walking part, participants were asked to walk on a treadmill. We used a treadmill for convenience due to the limited length of the wires, which connected the bands with the data acquisition hardware. Talking while walking usually occurs at slower speed, and therefore, we selected 2 mph for this test.

### 2.5. Participants

Fifteen young adults were asked to participate in this study. Participants were between 19 and 30 years old and were students at Simon Fraser University (SFU), Canada. Table 1 shows the participant characteristics. Written informed consent was obtained from all participants prior to data collection. The study was approved by the Research Ethics Board of SFU.

### 2.6. Talking Detection Algorithm

**Figure 3:**
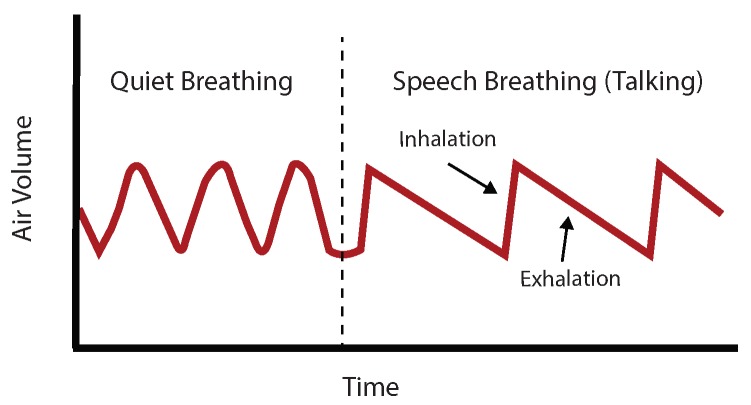
Changes in the air volume while talking.

Our main aim was to detect talking based on changes in the respiratory signals. Before talking, air usually gets inhaled fast and then exhaled slowly while talking. This results in a specific breathing pattern when compared to normal breathing (Figure 3). Our algorithm utilizes this information to detect talking.

**Figure 4:**
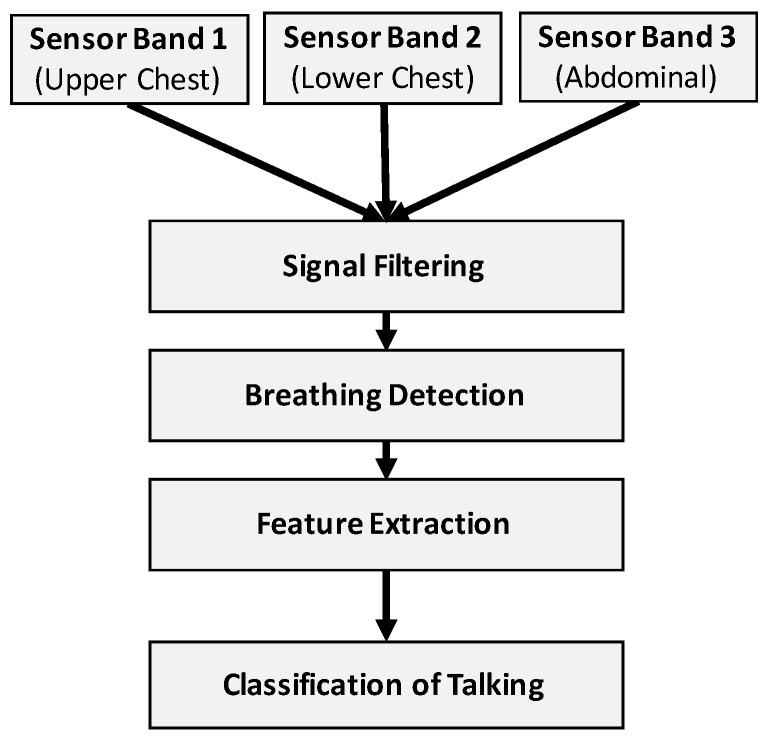
Design of the talking detection algorithm incorporating machine learning.

Our algorithm is based on the following steps of data processing and analysis (Figure 4):

- Data input: The input data to our algorithm were the raw sensor signals (sampled with 100 Hz) of the three bands, which we converted from voltage to resistance values.
- Signal filtering: A healthy adult usually breathes between 12 and 18 times per minute at rest. For older adults, the breathing can vary between 12 and 30 times per minute [27]. We filtered the sensor signals accordingly with a bandpass filter (4th order Butterworth, lower cut-off frequency of 0.1 Hz and higher cut-off of 1.5 Hz) to account for possible drift and reduce the overall level of noise in the sensor signals.
- Breathing detection: Any inhalation of air and consequent expansion of the torso results in a peak of the stretch sensor signal. Our algorithm detects these peaks using MATLAB’s peak detection algorithm with an empirically-defined parameter of 5 for the minimum peak prominence setting. The prominence of a peak measures how much the peak stands out due to its intrinsic height and its location relative to other peaks.
- Feature extraction: The detection of a peak triggers the feature extraction process. The algorithm centres a window with an empirically-found length of 3 s on each detected peak. From this time window, a set of predefined features get extracted and used as the input to a machine learning classifier.
- Classification of talking: A machine learning classifier has been trained to detect speech breathing (i.e., talking) based on the extracted features.

### 2.7. Machine Learning Approach

In the first part of the analysis, we were focused on identifying which machine learning algorithm, hyper parameters and features would generally perform best in the task of detecting talking using this kind of technology. In the second part of the analysis, we applied the selected model and calculated the accuracy for each participant.

#### 2.7.1. Model Selection

Four machine learning algorithms have been selected to investigate their feasibility in detecting talking based on our collected data. We have selected these four algorithms because they have been commonly used in health-related machine learning tasks and have achieved promising results in the past. First, random forest is an ensemble method that operates by constructing multiple decision trees at training time and then uses the mean prediction of individual trees to estimate the target values [28]. Second, neural network is a method inspired by the biological neural network system using layers and a number of interconnected nodes to make a prediction [29]. Third, support vector machine operates by constructing a set of hyperplanes in a high- or infinite-dimensional space to estimate the target value [30]. Fourth, linear discriminant analysis uses a linear decision boundary and has been proven to work well in practice due to its low computational costs [31].

The hyper parameters for the machine learning classifiers were empirically identified. To calculate the performance of each model and select the best performing hyper parameters, we used 15-fold cross-validation. This was done on a training dataset that consisted of the first 70% of data of each participant. The model performance was evaluated using the receiver operating characteristics curve (ROC) and the associated area under the curve (AUC) metric.

For the random forest classifier, the best performance was achieved using 200 as the parameter for the number of trees (values tested between 10 and 200). For the support vector machine, the best performance was achieved with gamma set to 0.01 (tested between 0.001 and 1) and C set to 10 (tested between 1 and 100). For the neural network classifier, the best performance was achieved with a network structure of 2 hidden layers (tested from 1 to 2) and 30 neurons in the hidden layers.

#### 2.7.2. Feature Extraction and Selection

Features were extracted with an automated feature extraction approach. Therefore, we used the Python library tsfresh [32], which calculates and tests more than 100 predefined time and frequency-domain features with various parameters. Using this approach, we extracted features from the raw and first derivate of the sensor signals of all three bands. Features were extracted using a sliding window (size of 3 s) approach. For the feature selection, we also applied 15-fold cross-validation and used the same training dataset as for the hyper parameter tuning. A tree-based approach was used to rank the best performing features based on their relevance (i.e., Gini importance [33]) for each run. Only the top 10% features among all runs were selected for the final algorithm to reduce complexity and computation time. For a detailed description of the calculation of these features, see [32]. The majority of significant features were based on the sensor signals of the upper chest and lower chest band. The features included in our final model were:

- Ratio beyond sigma: the ratio of values that are more than r×std(x) away from the mean of *x* (with r={1,2}).
- Symmetry looking: the Boolean variable denoting if the distribution of *x* looks symmetric.
- Continues Wavelet Transform peaks: the number of peaks of the continuous wavelet transform using a Mexican hat wavelet [34].
- Skewness: the sample skewness of *x* (calculated with the adjusted Fisher–Pearson standardized moment coefficient G1).
- Energy ratio by chunks: the sum of squares of chunk *i* out of *N* chunks expressed as a ratio with the sum of squares over the whole (with N=10).
- Augmented Dickey–Fuller: the hypothesis test that checks whether a unit root is present in *x* [35].
- Count above mean: the number of values in *x* that are higher than the mean of *x*.
- Count below mean: the number of values in *x* that are lower than the mean of *x*.
- Number of crossings: the number of crossings of *x* on *m* (with m=0).
- Fourier coefficients: the coefficients of the one-dimensional discrete Fourier transform [36].
- Welch’s spectral density: the cross power spectral density of *x* [37].
- Sample entropy: the sample entropy of *x*.
- Autoregressive coefficients: the fit of the unconditional maximum likelihood of an autoregressive AR(k) process.

#### 2.7.3. Performance Evaluation

We integrated the best performing machine learning model, features and parameters into our algorithm and evaluated its performance in detecting talking in an intra-subject analysis. The data of each participant were split into the activities of sitting, standing and walking. For each activity, we trained a model separately and evaluated it using cross-validation.

As sample-based performance metrics, accuracy (ACC), true positive rate (TPR) and false positive rate (FPR) were selected. TPR has been defined as the percentage of correctly identified speech breathing patterns. FPR has been defined as the percentage of incorrectly identified speech breathing patterns among all other breathing patterns.

(1) $$ACC=\frac{TP+TN}{TP+TN+FP+FN}$$

(2) $$TPR=\frac{TP}{TP+FN}$$

(3) $$FPR=\frac{FP}{FP+TN}$$

Furthermore, the number of correctly identified talking segments was calculated. A talking segment was classified correctly if the majority of prediction labels in this segment predicted talking.

(4) $$ACC_{seg}=\frac{correctly\_classified\_talking\_segments}{total\_number\_of\_talking\_segments}$$

### 2.8. Software

MATLAB (R2016b) was used for data acquisition, processing of the sensor data and algorithm development. The Python package scikit-learn [38] was used to train and evaluate the machine learning models. The Python package tsfresh [32] was used for automated feature extraction.

## 3. Results

**Figure 5:**
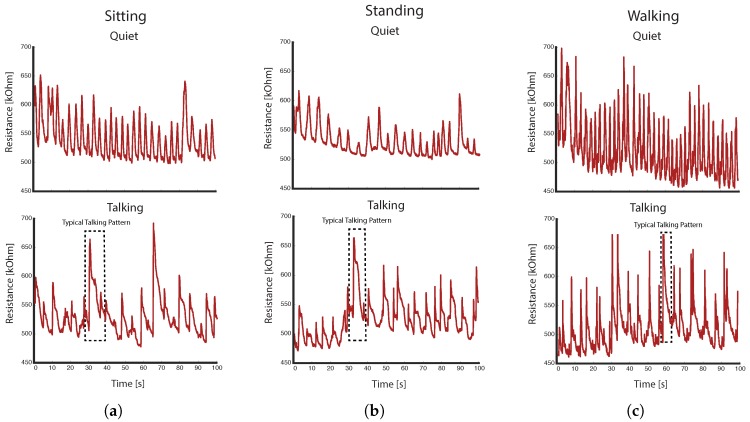
Comparison of the raw sensor signals (upper chest band) between quiet and speech breathing (i.e., talking) for: (**a**) sitting; (**b**) standing; and (**c**) walking.

One hour of sensor data was recorded from each participant with a recording time of 30 min of talking. The entire dataset included 11,924 detected breathings, which were used for further classification. We observed significant differences between normal and speech breathing in the activities of sitting, standing and walking (Figure 5). During the phases of talking, the breathing is less rhythmic with faster inhalations and slower exhalations.

### 3.1. Model Selection

**Figure 6:**
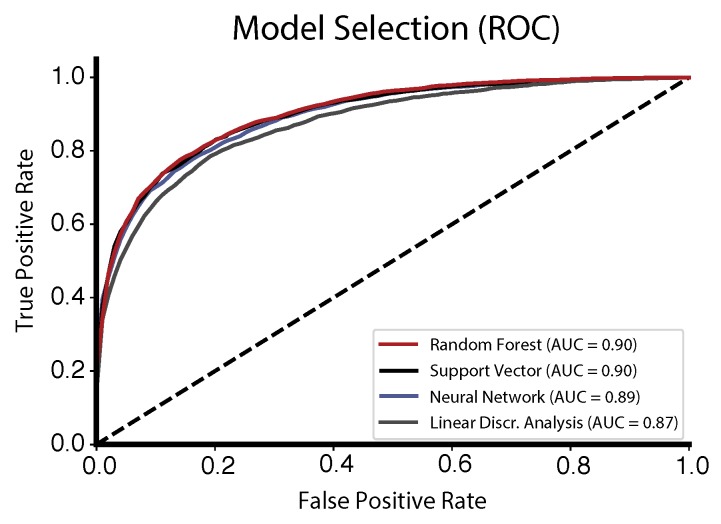
Comparison of the ROC curves (and the associated AUC metric) among the tested machine learning algorithms.

Among all tested machine learning algorithms, the random forest (and support vector machine) classifier performed best on our dataset with an AUC value of 0.90, which was slightly higher compared to the performance of the neural network classifier (AUC = 0.89) and linear discriminant analysis (AUC = 0.87) (Figure 6).

### 3.2. Accuracy of Talking Detection Algorithm

**Figure 7:**
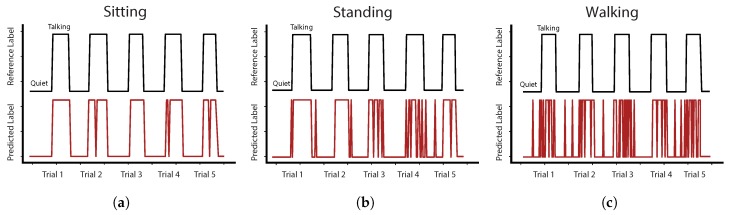
Exemplary detection of talking for participant P10 in activities: (**a**) sitting; (**b**) standing; and (**c**) walking.

Among all participants, our algorithm utilizing the random forest classifier detected talking with an average *ACC* of 85% (*TPR*: 81.3%, *FPR*: 12.8%) (Table 2). The highest *ACC* of 88% was achieved in the sitting task and the lowest *ACC* of 80.6% in walking. Table 3 shows the results for each participant in detail with the accuracy ranging from 68.8% to 97.5%. Furthermore, segments of talking have been correctly classified with an *ACC*${}_{seg}$ of 96.3%. Figure 7 illustrates the exemplary prediction accuracy of our algorithm on the data of participant P10. The number of misclassifications increased from sitting, standing to walking.

## 4. Discussion

We developed an algorithm that can detect if the user is talking based on respiratory markers. In contrast to previous work, we used textile-based stretch sensors to monitor the expansion and contraction of the torso and achieved a reasonable accuracy by incorporating machine learning into our algorithm.

Previous studies have relied on either audio or video recordings to detect talking. Besides the technical challenges of these approaches, there might be also privacy concerns [9]. The aim of this study was to develop a system that is unobtrusive and portable. We selected a wearable approach, as it would allow quantifying talking throughout the day independent of the user’s location. This is in alignment with a recent trend in the development of the wearable technologies for various health applications [39,40].

Our approach uses wearable textile-based sensors to monitor breathing and as a consequence detect if someone is talking. Although there were some studies that have investigated the feasibility of detecting respiratory events in the past, only a few studies have focused on the detection of talking in respiratory signals [10,12,14]. These studies have used inductive plethysmograph sensors. Conventional inductive plethysmograph sensors are primarily designed for the clinical setting and short-term recordings with possible limitations in the size of the electronics and number of sensors that can be used at the same time [41].

In terms of accuracy, Rahman et al. [10] (and Bari et al. [42]) reported 82 to 87% in speech/non-speech classification using inductive plethysmograph sensors. The reported accuracy is in alignment with what we have achieved in this study.

What differentiates this work is the use of textile-based stretch sensors in combination with the developed machine learning-based algorithm. The sensor we used is flexible with a diameter of only 0.4 mm and acts like a resistor, which makes it easy to integrate into garments and to acquire measurements. We proposed an algorithm suitable to detect talking including a comprehensive identified and discriminative set of features upon which future work can build.

What we have observed is that breathing and the corresponding patterns were quite heterogeneous between participants. Breathing was either shallow, normal or deep, and for some participants, the chest expansion was more noticeable, whereas for others, the abdominal region expanded more. We compensated for this behaviour by training our algorithm individually for each participant. In practice, this would suggest that a calibration phase might be needed before the system can be used by an individual. Another factor that might have influenced the accuracy was the sensitivity of the technology to noise due to body movements. Breathing and the corresponding expansion of the torso result in a relatively small elongation of the stretch sensor. What we have observed is that rotational and bending movements of the upper body influenced the measurements. This was especially noticeable in the task of walking, which might explain the lower accuracy in this task. Future work might combine our approach with an accelerometer to filter out the noise due to body movements.

Considering the advantages of the technology, this approach might be suitable for the daily life setting. A future application could be the integration of the sensor (or a series of sensors) into a tight-fitting undershirt. In addition to the sensor, a circuit board and battery would be required. Preliminary results show that the sensor has a power draw of about 1.25 mW (as used in this study). This would allow the monitoring of the user’s level of talking throughout the day, and furthermore, this measurement could be used as an indicator of social interaction. Such a system might be used in older adults where social isolation and loneliness are common concerns [1,3]. For example, in an institutionalized setting, such a system could provide the staff daily feedback about the level of social interaction of each resident. Once a significant change in behaviour has been detected, targeted interventions could be started. Similarly, this technology could be used in older people living in the community where a low level of social interaction can lead to more frequent home visits by the healthcare professionals.

We acknowledge certain study limitations. Data were collected in the laboratory setting under fairly controlled conditions with young and healthy adults. Participants were asked to read a text out loud, which might be different from conversational speaking. Future studies are warranted to determine whether this approach can be used in a daily life setting and to investigate the accuracy and user acceptance of this system in the older population.

In summary, we have demonstrated that wearable textile-based sensors in combination with a machine learning-based algorithm can be used to detect when the user is talking. In future, this approach may be used to unobtrusively quantify talking as an indicator of social interaction, and consequently may prevent social isolation and loneliness.

## Figures and Tables

**Table 1:** Participant characteristics.

	Study Participants (*n* = 15)
Age (years)	23 (3.8)
Gender (F/M)	6/9
Height (cm)	169.8 (8.9)
Weight (kg)	68.5 (12.1)
BMI (kg/m${}^{2}$)	23.6 (3.1)

**Table 2:** Average performance of our algorithm in detecting talking among all participants.

	Average *ACC*	Average *TPR*	Average *FPR*
**Sitting**	88.0 (5.4)	88.0 (6.1)	12.6 (6.9)
**Standing**	86.3 (7.3)	84.2 (8.6)	12.5 (7.6)
**Walking**	80.6 (7.7)	71.8 (12.1)	13.3 (6.5)
**Average**	85.0 (6.8)	81.3 (8.9)	12.8 (7.0)

**Table 3:** Performance results of our algorithm in detecting talking for each participant (P).

	P01			P02			P03			P04			P05		
	***ACC***	***TPR***	***FPR***	***ACC***	***TPR***	***FPR***	***ACC***	***TPR***	***FPR***	***ACC***	***TPR***	***FPR***	***ACC***	***TPR***	***FPR***
**Sitting**	94.3	92.3	3.9	94.8	93.5	4.2	97.5	94.9	0.9	84.9	84.9	15.0	82.9	81.2	15.6
**Standing**	93.9	92.2	4.4	90.5	90.0	9.1	94.2	91.5	3.7	76.1	73.1	21.3	86.0	79.1	9.9
**Walking**	79.6	76.4	17.6	85.2	81.0	11.6	90.2	82.4	4.4	68.8	55.3	20.7	87.2	83.6	9.8
	**P06**			**P07**			**P08**			**P09**			**P10**		
**Sitting**	81.6	87.7	24.2	88.6	95.1	20.2	93.7	93.1	5.9	89.3	93.3	14.6	92.0	90.9	7.3
**Standing**	82.5	79.2	14.7	94.0	94.9	7.1	95.1	92.5	2.9	93.6	96.1	8.9	87.0	81.4	9.1
**Walking**	71.1	70.5	28.5	87.8	84.7	9.3	95.6	90.8	1.4	79.7	75.4	17.0	81.1	68.2	11.9
	**P11**			**P12**			**P13**			**P14**			**P15**		
**Sitting**	82.7	82.0	16.7	89.8	87.0	7.9	83.5	89.3	22.7	84.3	79.1	12.6	79.3	75.0	16.7
**Standing**	73.9	73.8	26.1	83.3	70.8	9.0	78.7	76.2	18.8	75.8	79.5	28.2	89.8	93.2	13.9
**Walking**	68.3	42.5	14.8	79.4	69.4	13.5	73.0	61.3	18.6	81.7	68.2	9.9	80.0	66.9	9.9
